# Acute and Time-Course Effects of Traditional and Dynamic Warm-Up Routines in Young Elite Junior Tennis Players

**DOI:** 10.1371/journal.pone.0152790

**Published:** 2016-04-12

**Authors:** Francisco Ayala, Víctor Moreno-Pérez, Francisco J. Vera-Garcia, Manuel Moya, David Sanz-Rivas, Jaime Fernandez-Fernandez

**Affiliations:** 1 Sports Research Centre, Miguel Hernandez University, Elche, Spain; 2 Tennis Performance Research Group, Madrid, Spain; 3 Royal Spanish Tennis Federation (RFET), Madrid, Spain; Research Center for Sports Sciences, Health and Human Development (CIDESD), University of Trás-os-Montes e Alto Douro, Vila Real, Portugal, PORTUGAL

## Abstract

Despite the large number of studies that have examined the acute effects of different warm up modalities (WU) on physical performance, none of them have documented the time course of potential performance recovery in tennis players. The aim of this study was twofold: (a) to analyze and compare the acute effects of two different WU modalities (traditional WU [TWU] and dynamic WU [DWU]) on physical performance (i.e., CMJ, sprint, serve speed and accuracy) in elite junior players, as well as (b) to monitor the time course of any WU-induced changes after 30 and 60 min of simulated match-play. Twelve junior elite players completed both WUs modalities (TWU and DWU) in a counterbalanced order on separate days. In each experimental session, counter movement jump (CMJ), 20-m sprint, tennis serve speed and accuracy tests were performed before (immediately after TWU or DWU) during (30 min) and after 60 min of a simulated match play. Measures were compared via four factorial (WU intervention and time) repeated measures ANOVAs. There were main effects of WU (TWU and DWU) throughout the time for all the variables analysed. The results indicate that DWU routine led to significantly faster 20 m sprint times and higher CMJs as well as faster and more accurate tennis serves at both post warm-up and 30 min match-play testing moments in comparison with the scores reported by the TWU routine (p < 0.05; positive effects with a probability of >75–99%). No significant intergroup differences were found at 60-min match-play testing moment in any variable (except for the 20 m sprint). Therefore, the findings of this study recommend for optimal performance in these elite tennis players, DWU routines should be performed prior to formal training and competition rather than TWU routines.

## Introduction

Tennis has experienced a significant increase in popularity in recent years, with more than 75 million people participating both, at recreational or professional levels [1]. Like almost all athletes, tennis players also perform warm-up routines (WU) prior to formal training and competition. Traditionally, and similar to other sports, tennis-specific WUs have included some active aerobic activities (including running, light calisthenics), static stretching exercises (SS) of the major muscle groups and sport-specific movements incorporating various range of motion exercises with skills-based drills executed at, or just below game intensity [2].

Low to moderate intensity aerobic activity is an important element of the WU, increasing muscle temperature which is directly responsible for a number of mechanisms important for short term performance (i.e., range of movement in the joints, increased rate of nerve impulses) [3]. In contrast, the SS component of traditional WU (TWU) routines in many sports, including tennis, has demonstrated an acute negative effect on isolate physical performance variables (e.g. vertical jump, sprint, agility) [4, 5]. However, it is still unknown whether the negative effect of SS on maximal muscular performance might remain even when followed by additional sport-specific WU component. In this regard, whereas some studies have reported that the sport-specific component of the TWU attenuated the negative effect of SS on maximal muscular performance [6–9], other studies have not reported any damped effect of the sport-specific component [10–12].

Parallel to the study of the effects of SS on force and power production, researchers have studied the use of so-called dynamic stretching exercises (DS) in WUs. The DS are basically active aerobic activities combined with sport-specific whole body movements that have been designed to be an extension of the general WU, under the term of “dynamic warm-up” (DWU). In contrast to TWU, evidence exists indicating that DWU induce improvements in strength and power performance [13]. Although the exact mechanisms by which DWU may improve strength and power performance are not well known, previous studies have found that DWU exert positive effects on muscular performance via contraction history dependent neuromuscular factors like post-activation potentiation (PAP) and stretch-shortening cycle (SSC) [14–18]. PAP is a phenomenon by which muscular performance is acutely enhanced when preceded by maximal or near maximal neuromuscular activation [19]. Two mechanisms responsible for PAP induced by DWU have been proposed: a) an increase in the phosphorylation of myosin regulatory light chains [20]; and b) increased recruitment of higher order motor units [21]. In addition, it has been suggested that DWU may induce a positive effect on the SSC via a greater action potential of the myotatic reflex that may result from the high stretching speed generated during the dynamic movements performed [6]. Some studies have examined whether the use of light (ranging from 2% to 15% of body weight) [16, 22–24] and heavy (ranging from 45% to 90% one repetition maximum) [25–27] external resistances (e.g. dumbbells, weight vests or barbells) during the DWU could amplify the maximal muscular performance to a greater degree than DWU without external resistances. Several of the above-mentioned studies [22–24], although not all [16], have found that the use of light external resistances coupled with DWU may not be effective in increasing performance-benefits of DWU through these neuromuscular factors. Contrary to the use of light external resistances, some studies have reported that the use of heavy external residences coupled with DWU may elicit an enhancement in sports performance measures (i.e. jumping height, sprinting times) in a greater extend than DWU without external resistances through these above-mentioned contraction history dependent neuromuscular factors (mainly PAP) [25–27]. However, further studies are necessary to confirm these findings and demonstrate theirs practical applications on different sport-related contexts.

These different effects of the TWU and DWU on maximal muscular performance, along with the fact that both WU modalities have demonstrated similar effectiveness to improve joint range of motion [11, 28], have led some International Sports and Fitness Organizations (included the International Tennis Federation [ITF]) to recommend that the TWU should be replaced by DWU prior to any sport events [29].

However, analysing the body of literature regarding the acute effects of the TWU and DWU on sports performance, some limitations are noted, which should be clarified before recommendations to athletes can be made. For instance, a recent meta-analysis regarding the acute effects of pre-participation SS on muscle performance has pointed out that some of the studies have used overall stretch durations on a single muscle group (i.e., quadriceps, gastrocnemius and hamstrings mainly), ranging from 90s to over 8 min [5]. These SS doses per single muscle group are not representative of TWUs used by athletes (including tennis players) to prepare themselves for exercise or competition. Another aspect that should be highlighted is that despite the large number of studies that examined the acute effects of the DWU on muscle performance [6, 7, 9–11, 14–16, 22–24, 30–33], very few of them have documented the time course of potential performance recovery using non-athletes [34] and football players [17] as participants. Previous studies have reported that there is a significant inter-individual variability in the PAP phenomenon, so that stronger individuals are able to express higher levels of PAP [20]. Furthermore, the total work or load performed during the DWU might also influence the voluntary PAP response because too little work may not trigger the mechanism(s) responsible for PAP whereas too much work may induce high levels of fatigue, thus masking the potentiation effects [17]. Consequently, the study of the length of time that any DWU-induced improvement in muscle performance persists should be carried out in athletes with similar sport background (sport modality, level of performance, sex, years of practise) and using sport-specific DWUs in order to minimise the effects of the different individual’s strength levels and WU loads on the PAP response. Consequently, the length of time that any DWU-induced improvement in muscle performance persists in tennis players is not well known.

In addition, most of the studies that examined the time course of the dose response effects of the TWU on muscle performance [35–39], although not all [40], are limited by: (a) the sport-decontextualized SS protocols employed (long single muscle group stretch durations [ranged from 3 min to 60 min]; (b) single-muscle protocols [mainly hamstrings or plantar flexors]); and (c) muscular performance tests selected (mainly isometric and isokinetic strength). Finally, only two studies have analyzed the acute effects of TWU and DWU in tennis players [31, 38]. Both studies found that adding SS on the general 5-min WU (including active aerobic activities and practise serves and groundstrokes) did not affect serve speed. Furthermore, Gelen et al. [31] reported that the use of DS as part of the above-mentioned general WU produced an increase of 1–3% in tennis serve ball speed over WU alone. However, the effect of incorporating a tennis-specific skills and movement patterns component after the SS and active aerobic activities components on serve speed was not assessed in neither of them. In addition, apart from the serve speed, no other tennis-specific performance measures (i.e., sprint, jumping height) were assessed.

Therefore, there is a clear need for applied research studies that analyse and compare the acute and retentive effects of realistic TWU and DWU protocols on sports-specific performance measures. The knowledge about which pre-participation WU modality (TWU or DWU) can optimize better the maximal muscle performance seems to be essential for tennis players, as they must perform high-intensity short-term motor activities (i.e., accelerations, serves and groundstrokes) since the beginning until the end of a match [41].

Therefore, the aim of the present study was twofold: (a) to analyse and compare the acute effects of two different WU modalities (TWU and DWU) on physical performance (i.e., jumping, sprint, serve speed and accuracy) in elite junior tennis players; and (b) to monitor the time course of any WU-induced changes after 30 and 60 min of simulated match-play.

## Materials and Methods

### Participants

Twelve internationally ranked (top 100 ITF Juniors rankings) male junior tennis players (mean ± SD: age = 16.8 ± 0.3 years; body mass = 73.4 ± 6.4 kg; stature = 182.1 ± 3.2 cm) completed this study. All the players participated on average in 14–16 h of training (i.e., on-court) per week, plus 3.2 ± 0.4 matches per week, including singles and doubles. None of the participants reported any current or ongoing neuromuscular diseases or musculoskeletal injuries and none of them were taking any dietary or performance supplements that might be expected to affect performance during the study. Before any participation, experimental procedures and potential risks were explained fully to the participants, and a written informed consent was obtained from them and their parents/guardians. The Institutional Research Ethics committee (University Miguel Hernandez of Elche [Spain]), conformed to the recommendations of the Declaration of Helsinki, specifically approved this study.

### Experimental design

The study took place at the beginning of the summer competition season (April–May). A counter-balanced crossover-study design, in which participants performed all interventions, was used. Participants visited our laboratory on three occasions, with 2-weeks rest between sessions. The first visit was a familiarisation session, including the different testing procedures and WU exercises, and the following two visits were the experimental sessions.

**Fig 1:**
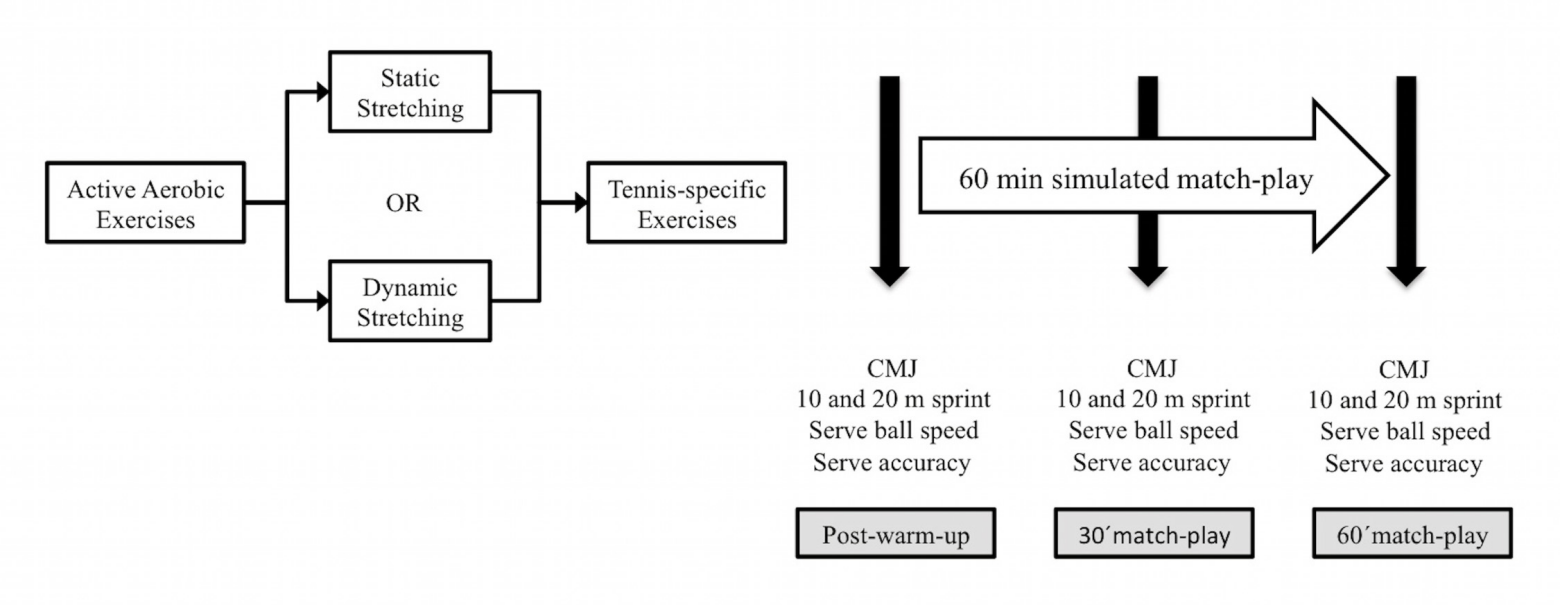
Schematic representation of the intervention.

During each experimental session, participants began by completing one of the two interventions: TWU or DWU, which were counterbalanced per person to avoid carryover effects. The assessments of the (a) jumping height, (b) 20 m sprint time, (c) serve speed and (d) accuracy were carried out before (post-warm-up), during (30´match-play) and after (60´match-play) a 60 min simulated tennis match-play. The order of the tests was consistent through the experimental sessions and is displayed in Fig 1. Simulated matches were played consecutively.

To reduce the interference of uncontrolled variables, all the participants were instructed to maintain their habitual lifestyle and normal dietary intake before and during the study. The participants were told not to exercise on the day before a test and to consume their last (caffeine-free) meal at least 3 hours before the scheduled test time.

### Warm-Up

Each WU intervention lasted about 15–20 min. Aside from the stretching component, each WU followed the exact procedure in both experimental sessions (TWU and DWU), consisting of the following given below. Participants started performing at least a 4–5 min of self-paced general WU consisting of low- to moderate-intensity (self-perceived) running including forward/backwards movements, sidestepping and general mobilization (i.e., arm circles, leg kicks), followed by 10 minutes of a designated stretching routine (SS for TWU or DS for DWU) before completing 6–8 min of tennis-specific intensity activities (e.g., on-court hitting against an opponent performing ground strokes, volleys and serves). Following these stretching routines, participants performed 3 sets of ballistic exercises with a 15 s rest period between each set. Exercises included single hop jumps (5 repetitions), alternate leg bounds (multidirectional x 5 repetitions), service motion throws without a tennis ball (5 repetitions each arm) and short (2–3 m) accelerations and decelerations in different directions (3 repetitions forwards and 5 repetitions side to side).

The rationale of using this WU structure (general aerobic activities + stretching exercises + tennis-specific activities) was to replicate the WU structure that is recommended by the ITF and usually performed by tennis players [42].

#### Dynamic stretching

Participants performed 6–8 min of DS (i.e., straight leg march, forward lunge with opposite arm reach, forward lunge with an elbow instep, lateral lunge, trunk rotations, multidirectional skippings) performing 3 sets, from low to high intensity, with a 15 s rest period between each set. Each exercise was performed in a controlled manner through a range of motion required in many sports [16].

#### Static Stretching

The SS routine consisted of exercises designed to stretch the plantar flexors (principally gastrocnemius and soleus), hip flexors (hamstrings), hip extensors (gluteals), hip adductors, quadriceps, posterior shoulder, triceps, shoulder external, pectoralis, deltoid, biceps brachii, and forearm extensors and flexors. Exercises were selected based on previous literature [6, 7, 18] and performed in similar order. Exercises were repeated 2 times and were performed for 30 s since this is the minimum dosage that has been associated with significant acute decreases in maximum muscular strength and power [5, 43]. A 10-s recovery period between each exercise was given. No rest period was allowed when changing the limb. Stretching intensity was held at the point of discomfort.

### Vertical Jumping

A CMJ without arm swing was performed on a contact platform (Ergojump®, Finland) according to Bosco et al. [44]. During the CMJ, the participants first stood upright, then squatted to a self-selected depth of approximately 90° knee flexion, and jumped immediately as high as possible. Players were asked to keep their hands on their hips to prevent the influence of arm movements on vertical jump performance. In addition, players were allowed to perform a countermovement with the lower limbs before jumping.

Each player performed 2 maximal CMJs interspersed with 45 s of passive recovery, and the best height for each was recorded.

### 20-m sprint

Owing to its good reproducibility, linear sprint tests ranging from 10 to 30 m are used as general measures of linear acceleration and speed [45]. Time during a 20-m dash in a straight line was measured by means of single beam photocell gates placed 1.0 m above the ground level (Time It; Eleiko Sport, Halmstad, Sweden). Each sprint was initiated from an individually chosen standing position, 50 cm behind the photocell gate, which started a digital timer. Each player performed 2 maximal 20-m sprints interspersed with 3 min of passive recovery, and the fastest time achieved was retained.

### Serve speed

A radar gun (Stalker Professional Sports Radar, Plymouth, MN, USA) was used to measure serve speed, following the methods previously described [46]. Before each experimental session, the radar gun was calibrated in accordance with the manufacturer’s specifications. The radar was positioned on the center of the baseline, 4 m behind the server, aligned with the approximate height of ball contact (~ 2.2 m) and pointing down the center of the court. Players performed 5 maximum effort serves with approximate the same spin (flat or slice) to the "advantage" service box. To be recorded, serves had to be in the service box. The highest speed recorded was used for analysis.

### Serve Accuracy

For serve accuracy, players performed another 5 maximum effort serves with approximate the same spin (flat or slice) to the "advantage" service box. The accuracy scores were determined by counting the number of times the ball landed within a designated target perimeter. Target dimensions were designed considering similar methodologies, available resources, through discussion with coaches and athletes and preliminary trials [47]. The target area for the serve (1.8 × 1.8 m) was inside the intersection of the service line and the centre line. Participants served from the deuce court and were instructed to “serve first serves flat and down the T” (centre line). Shots landing within target areas were ranked according to a 3, 2, 1, scoring system. Balls landing outside the perimeter of the target areas (i.e., errors) received a 0 score. A total score, expressed as a percentage of the maximum, was recorded for each trial.

### Statistical analysis

Before data collection, the intra and inter-session reliability of the jumping height, 20 m sprint time, serve speed and accuracy measures was determined using a test-retest design [48]. Thus, the same testing procedure that was carried out in the current study was repeated twice at 5-day intervals in 7 elite tennis players, who were not included in the current study. Intra and inter-session intraclass correlation coefficients (ICC_2k_) and coefficients of variation (typical error of measurement expressed as a percentage [CV]) were calculated for each measure using the method previously described by Hopkins [49].

Means ± standard deviations (SD) were used to describe variables. Data normality and homoscedasticity were confirmed before inferential analysis through Kolmogorov-Smirnov and Levene’s test, respectively.

Four factorial (WU intervention and time) repeated measures ANOVAs were used to analyse and compare TWU and DWU responses for each of the three testing moments (post-warm-up vs 30´match-play; post-warm-up vs 60´match-play; 30´match-play vs 60´match-play). Each participant’s change score was expressed as a percentage of baseline score (post-warm-up) via analysis of log-transformed values, to reduce bias arising from non-uniformity of error. Errors of measurement and individual responses expressed as coefficients of variation were also estimated. In addition, the analysis determines the chances that the true effects were medium, substantial or trivial when a value for the smallest worthwhile change is entered. Thus, the intra and inter-session CV determined during the pilot study was considered the smallest substantial/worthwhile change for each of the variables for intra and inter-groups comparisons respectively. The experiment-wise type I error rate was set at p < 0.05 and protected by adjusting the critical p values for each RMANOVA using a Holms correction.

The qualitative descriptors proposed by Batterham & Hopkins [50] were used to interpret the probabilities (clinical inferences based on threshold chances of harm and benefit of 0.5% and 25%) that the true affects are harmful, trivial or beneficial: <1%, almost certainly not; 1–4%, very unlikely; 5–24%, unlikely or probably not; 25–74%, possibly or may be; 75–94%, likely or probably; 95–99%, very likely; >99%, almost certainly. Effect sizes (ES) were also calculated to determine the magnitude of differences between the groups or experimental conditions for each variable using the method previously described by Cohen [51] (i.e., ES of ≤0.4 = small; 0.41–0.7 = moderate; >0.7 = large magnitudes of change, respectively).

## Results

### Reliability

Excellent intra- and inter-session reliability scores were demonstrated for all the performance measures analysed (ICC > 0.80 and CV < 5–10%) [37]. The intra and inter-session CV for each performance measure ranged from 1.5% to 4.9% and from 1.7% to 5.1% respectively, with ICC scores higher than 0.85 in both cases (Table 1).

**Table 1:** Intra e inter-sessions reliability statistics (typical percentage error [CV_TE_] and intraclass correlation coefficient [ICC_2,k_]) for each performance measure.

Measure	Intra-session reliability		Inter-session reliability	
	ICC_2k_	CV_TE_	ICC_2k_	CV_TE_
CMJ height	0.96 (from 0.93 to 0.98)	1.9 (from 1.2 to 3.4)	0.95 (from 0.93 to 0.97)	2.1 (from 1.2 to 3.7)
20 m sprint time	0.92 (from 0.90 to 0.93)	1.5 (from 0.5 to 2.2)	0.91 (from 0.89 to 0.93)	1.7 (from 0.2 to 2.5)
Serve speed	0.92 (from 0.90 to 0.95)	2.8 (from 1.3 to 6.0)	0.92 (from 0.91 to 0.94)	3.3 (from 1.1 to 6.7)
Serve accuracy	0.90 (from 0.68 to 0.93)	4.9 (from 1.3 to 7.9)	0.87 (from 0.82 to 0.91)	5.1 (from 1.4 to 8.7)

CMJ: countermovement jump; m: meters

### Acute Effects of Warm-up Protocols

Table 2 shows the mean and standard deviation for the physical qualities analysed among testing moments and separated by experimental conditions (TWU and DWU). The inter-group comparisons for each variable in all testing moments are also displayed in Table 2. Thus, the DWU routine reported significantly faster 20 m sprint times, higher CMJs as well as faster and more accurate tennis serves scores at both post WU and 30 min match-play testing moments in comparison with the scores reported by the TWU routine (p < 0.05; negative effects with a probability of >75–99%; d = 0.20–1.03). The highest differences were reported at post-warm up testing moment for all the variables analysed (p < 0.05; % changes ranged from 2.8 to 11%). In both testing moments, the serve speed and accuracy measures showed the highest differences (%e changes = 2.8–4.0% [p < 0.05; likely negative with a probability of >75–95%; d = 0.60–1.03] and 4–11% [p < 0.05; likely negative effects with a probability of >75–95%; d = 0.29–0.83] respectively), whereas the lowest differences were reported for the 20 m sprint time (% changes = 1.6–3.9% [p < 0.05; possible positive effects with a probability of >75–95%]). No significant inter-group differences were found at 60-min match-play testing moment in any variable (except for the 20 m sprint time values).

**Table 2:** Jumping height, 10 and 20 m sprint times, serve speed and serve accuracy descriptive statistic and mean percentage changes between stretching conditions (static versus dynamic) at each of the three testing moments (post warm-up, 30´match-play and 60´match-play). Qualitative inference and likelihood (%) of being positive/ trivial / negative of the effects are also shown.

Testing moments	Static stretching	Dynamic stretching	% Change (Mean ±90 CL)	Effect Size (d)	Chances that the true effects were substantial^a^ (%)			
					Positive	Trivial	Negative	Qualitative inference^b^
**Post warm-up**								
Jumping height (cm)	41.5 ±3.3	42.7 ± 3.3	-2.8 (-3.9 to 1.7)	-0.35	88	12	0	Likely positive
20 m sprint time (s)^t^	3.11 ±0.11	2.99 ±0.10	3.9 (2.8 to 4.9)	1.06	100	0	0	Most likely positive
Serve speed (km/h)	181.2 ±6.9	188.7 ±6.9	-4.0 (-5.1 to -2.9)	-1.03	88	12	0	Likely positive
Serve accuracy*	53.8 ±8.1	60.1 ±8.1	-11 (-17.4 to -4.2)	-0.83	99	1	0	Very likely positive
**30´match-play**								
Jumping height (cm)	41.3 ±3.2	42.7 ± 3.3	-3.3 (-4.3 to 2.2)	-0.40	96	4	0	Very likely positive
20 m sprint time (s)^t^	3.04 ±0.08	3.00 ±0.10	1.6 (1.0 to 2.3)	0.43	43	57	0	Possible negative
Serve speed (km/h)	182.7 ±5.6	188.0 ±7.1	-2.8 (-3.7 to -1.8)	-0.68	34	66	0	Possible positive
Serve accuracy*	63.5 ±6.6	66.6 ±9.2	-4.3 (-9.6 to 1.3)	-0.29	66	32	2	Possible positive
**60´match-play**								
Jumping height (cm)	42.2 ±3.9	42.4 ±3.9	-0.3 (-0.6 to 0.0)	-0.03	0	100	0	Most likely trivial
20 m sprint time (s) ^t^	3.04 ±0.08	3.00 ±0.11	1.4 (0.7 to 2.1)	0.35	21	79	0	Possible positive
Serve speed (km/h)	188.2 ±7.1	188.4 ±6.5	-0.1 (-0.6 to 0.4)	-0.03	0	100	0	Most likely trivial
Serve accuracy*	62.1 ±8.2	64.5 ±8.4	-3.7 (-9.3 to 2.3)	-0.26	15	85	0	Likely trivial

CL: confidence limits

*: The total score expressed as a percentage of the maximum (100%).

± 90% CL: add and subtract this number to the mean effect to obtain the 90% confidence limits for the true difference.

a Substantial is an absolute change in performance of > 2.1%, 1.7%, 3.3% and 5.1% for measures of jumping height, sprint time (10 and 20 m), serve speed, serve accuracy and serve score respectively for passing accuracy (see Methods).

b If chance of benefit and harm both >5%, true effect was assessed as unclear (could be beneficial or harmful). Otherwise, chances of benefit or harm were assessed as follows: <1%, almost certainly not; 1–5%, very unlikely; > 5–25%, unlikely; >25–75%, possible; >75–95%, likely; >95–99%, very likely; >99%, almost certain.

^t^: For the 20 m sprint time variable, a negative effect must be considered as an increase in the sprint performance.

### Time-Course of Warm-up Effects

**Fig 2:**
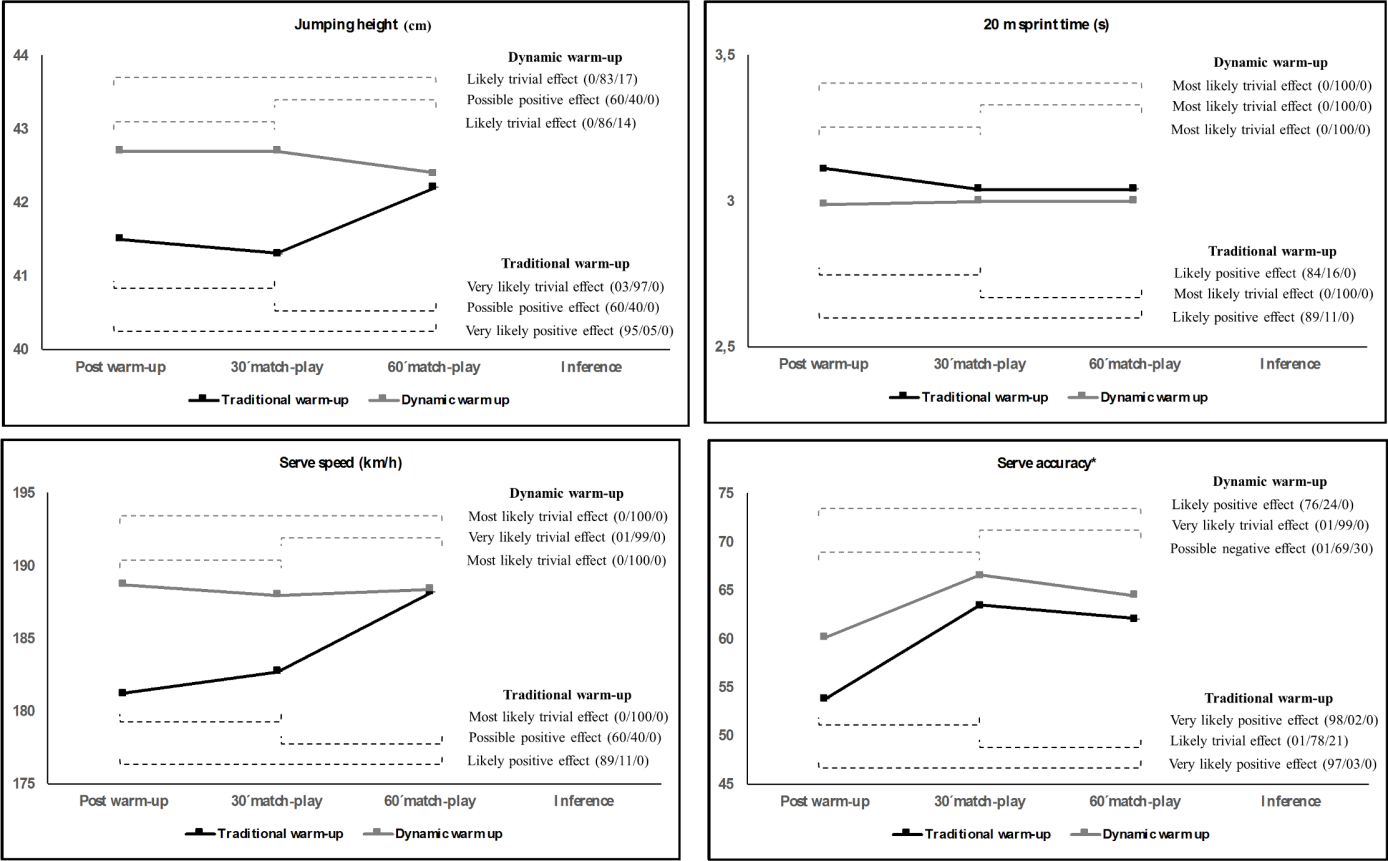
Time-course of warm-up effects. *: The total score expressed as a percentage of the maximum (100%).

Effects of TWU and DWU routines on CMJ, 20 m sprint time, serve speed and accuracy throughout the three different testing moments are presented in Fig 2. For the TWU routine, no meaningful differences (when the chances of benefit or harm were considered at least “likely” with a probability of >75–95%) were found for paired comparisons between consecutive testing sessions (30´match-play vs Post-WU; 60´match-play vs 30´match-play) in all the variables analysed (except for the 20 m sprint time and serve score variables in the 30´match-play vs Post-WU paired comparison). However, there were significant differences in all variables between the post-WU and 60 min match-play testing moments so that better scores were reported at 60 min match-play testing moment (p < 0.05; main effects with a probability of >75–95%; d = 0.33–0.91). For the DWU, no significant differences were found for paired comparisons between consecutive (30´match-play vs Post-WU; 60´match-play vs 30´match-play) and non-consecutive (60´match-play vs Post-WU) testing sessions in CMJ, 20 m sprint time and serve speed values. However, a likely positive effect was found between the 30´match-play and Post-WU testing moments and between post-warm-up and 60 min match-play testing moments for the serve accuracy scores.

## Discussion

The primary findings of the present study showed that performing a DWU routine including active aerobic activities, DS and tennis-specific skills led to higher jumping heights, faster sprint times as well as superior serve performance compared with a TWU routine with SS followed by tennis-specific skills. In addition, the results also showed that the differences between interventions (i.e., TWU vs. DWU) appeared to be more evident immediately after the WU (change: from 3.9% to 11% [very likely negative]; d: from 0.49 [small] to 1.1 [large]) and were gradually reducing their magnitude throughout the simulated match-play until disappearing at 60 min of the game. Although from a statistical point of view the magnitude of these differences could be considered small in almost all performance measures (i.e. % change < 5%, except for serve accuracy variable measured post warm up), these changes could be considered as relevant for tennis performance. Since margins between winning and losing in tennis are very small, fractions of a second in a short sprint prior to the ball hitting or loss of centimetres in jump height during a service may be essential components of tennis tasks and contribute to competent performances [52].

Our findings are not consistent with the few previous studies that have investigated the acute effects of different WU modalities (i.e. TWU and DWU) on specific explosive qualities (sprint and jumping), showing that practice attempts of the required tasks or sports specific dynamic movements may offset some of the potential negative effects of SS when included prior to dynamic movements [6–9, 53]. These studies have suggested that this “damping phenomenon” could be due to the fact that the sport-specific dynamic movement’s component of the WU included activities with very similar neuromuscular and energetic requirements to those needed to successfully perform in the tests, which might have helped to mask any likely negative effects from the SS component. A possible explanation for these conflicting results could be attributed to the amount of SS performed. Although methodologies are consistent in the use of short overall SS durations per muscle group (< 60s), the present study included SS for both the upper and lower extremities in an attempt to accurately reflect a typical tennis-specific WU, in contrast to previous studies that only stretched the muscles located on the lower extremity [6–9, 17, 53]. An acute bout of SS may reduce muscle activation via peripheral (autogenic inhibition of the Golgi tendon reflex, mechanoreceptor and nociceptor afferent inhibition) and central nervous system (supraspinal fatigue) mechanisms, not only in the stretched muscle but also in the un-stretched contralateral muscle (via central nervous system mechanism) [35, 54]. Therefore, the higher stretching stimuli applied to the central nervous system in our study might have been large enough to produce transitory alterations in the supraspinal mechanisms that could have not been totally compensated by the sport-specific dynamic movements component, explaining the conflicting results found.

Another important finding of the current study is that the physical performance measures obtained by the DWU intervention were stable throughout the 60 min of simulated match-play (linear tendency), suggesting a transitory negative effect of the TWU routine on performance. As the current study design did not contemplate a control group, this last-mentioned suggestion should be considered as a hypothesis based on the previously reported literature results.

Transitory negative effects of the SS on muscular performance have also been reported in previously studies. For example, Mizuno et al. [39] found that static 5-min stretching caused an immediate decrease in maximal isometric plantar flexor strength that lasted for 10 min. Fowles et al [34] observed significant reductions in plantar flexor strength that continued for 60 minutes after termination of the 30-min stretch protocol. The results of the current study in conjunction with those previously reported suggest that the deficits of SS on maximal muscular performance are disabled in a 30–60 min time range after stretching. Therefore, whether SS is essential before a tennis match-play (some athletes might feel less ready when SS is omitted from the WU), coaches and players should ensure that stretching occurs at least 30 min before the match. A number of mechanisms have been suggested to explain this performance decreases associated with SS interventions, including alterations in the mechanical components of skeletal muscle contraction [55], decreases in muscle activation [13, 54]; or a combination of both, mechanical and neural factors [56].

Serve performance measures (speed and accuracy) showed the largest percentage reductions after the TWU routine (4 and 11% for speed and accuracy, respectively) and at 30 min of match-play (3 and 4% respectively), compared to the DWU. These findings have very important implications regarding the design of effective WU strategies in tennis, as the serve has been considered the most stable and predictable measure of on-court tennis performance and therefore, essential to achieve high levels of performance [32]. Present results are not consistent with previous research conducted with moderate trained tennis players (teenage and college players) showing that SS routines had no acute effect on serve performance when carried out before aerobic exercises and without the inclusion of any sport-specific skill activities after [31, 38]. These discrepancies may be related, apart from the obvious differences in the WU design, to the different SS volume. While the current study stretched the major muscle groups of the upper and lower extremities, the previous studies only stretched the muscles of the upper extremity, which might have the same implications to the central nervous system previously mentioned. Nevertheless, negative or no-effects are not positive evidence for including SS in preparation for high-level tennis serve performance, a clear argument that there is no prospective evidence of benefit to include SS in preparation for tennis in most players.

To the best of our knowledge this is the first study the time-interval of the negative effects of a TWU intervention on the physical performance of elite junior tennis players in a simulated match-play situation, and unfortunately, comparisons are not possible.

Although this is the first study analysing the acute effects of two different WU modalities (TWU and DWU) on physical performance in elite junior players before, during and after 60 min of simulated match-play, some limitations should be noted. The study did not analyse the effects of the pre-participation WU designed for this study without the stretching component, we are not able to know if the DS component by itself could have had any positive effect on the physical performance. However, previous studies have reported significant improvements in jumping height, sprinting times and serve speed when a WU with a DS component was compared to a WU without any stretch component [7, 30, 31]. Another limitation is the small sample size given the participants were elite players. In addition, the amount and intensity of the movements (i.e. lateral, jumping, cuttings manoeuvres) and groundstrokes performed by the players in each simulated match-play were not registered and perhaps the dynamic nature of a match-play may have biased the results. However, we tried to minimize the possible effect of the dynamic nature of the match-played on the results through randomizing the order of the intervention and by using a crossover design.

In conclusion, the findings of the present study showed that the use of DS as a part of tennis-specific WU results in superior performance levels (i.e., jump, sprint time, serve speed and accuracy) before and during a 60 min simulated match-play, compared to the same WU routine with SS instead of DS in these elite junior male tennis players. In addition, the results found that these negative effects of using SS routines after a tennis-specific WU can last for almost 30 min of a simulated competitive situation.

## Practical Applications

In young elite tennis players, a 15 min WU routine, combining a cardiovascular activation followed by DS and tennis-specific skills exercises, should be recommended in order to achieve high specific performance levels (i.e., serve speed). It seems important to suggest that, especially at young ages, coaches and strength and conditioning specialists should educate the players in order to be able to distinguishing between the routine used for improving flexibility (i.e., SS during the training sessions, matches, etc.) and the one used as part of the WU process, targeting to activate the muscle groups involved in a specific performance task [30]. Moreover, the use of different WUs with DS routines, combining different volumes (e.g., 2–3 sets) and intensities, as well as athletes of different levels (e.g., under U14 vs. U16) warrants future studies.

## References

[1] Pluim BM, Miller S, Dines D, Renström PA, Windler G, Norris B, et al Sport science and medicine in tennis. Br J Sports Med. 2007;41(11):703–4. 17957002 10.1136/bjsm.2007.040865 PMC2465261

[2] Edwards DA, Kurlander LS. Women's intercollegiate volleyball and tennis: Effects of warm-up, competition, and practice on saliva levels of cortisol and testosterone. Horm Behav. 2010;58(4):606–13. 10.1016/j.yhbeh.2010.06.015 20615408

[3] Bishop D. Warm up I: potential mechanisms and the effects of passive warm up on exercise performance. Sports Med. 2003;33(6):439–54. 12744717 10.2165/00007256-200333060-00005

[4] Kay AD, Blazevich AJ. Effect of acute static stretch on maximal muscle performance: a systematic review. Med Sci Sports Exerc. 2012;44(1):154–64. 10.1249/MSS.0b013e318225cb27 21659901

[5] Simic L, Sarabon N, Markovic G. Does pre‐exercise static stretching inhibit maximal muscular performance? Scand J Med Sci Sports. 2013;23(2):131–48. 10.1111/j.1600-0838.2012.01444.x 22316148

[6] Chaouachi A, Castagna C, Chtara M, Brughelli M, Turki O, Galy O, et al Effect of warm-ups involving static or dynamic stretching on agility, sprinting, and jumping performance in trained individuals. J Strength Cond Res. 2010;24(8):2001–11. 10.1519/JSC.0b013e3181aeb181 19855310

[7] Little T, Williams AG. Effects of differential stretching protocols during warm-ups on high-speed motor capacities in professional soccer players. J Strength Cond Res. 2006;20(1):203–07. 16503682 10.1519/R-16944.1

[8] Taylor KL, Sheppard JM, Lee H, Plummer N. Negative effect of static stretching restored when combined with a sport specific warm-up component. J Sci Med Sport. 2009;12(6):657–61. 10.1016/j.jsams.2008.04.004 18768355

[9] Bishop D, Middleton G. Effects of static stretching following a dynamic warm-up on speed, agility and power. J Hum Sport Exerc. 2013;8(2);391–400.

[10] Carvalho FL, Carvalho MC, Simao R, Gomes TM, Costa PB, Neto LB, et al Acute effects of a warm-up including active, passive, and dynamic stretching on vertical jump performance. J Strength Cond Res. 2012;26(9):2447–52. 22067244 10.1519/JSC.0b013e31823f2b36

[11] Perrier ET, Pavol MJ, Hoffman MA. The acute effects of a warm-up including static or dynamic stretching on countermovement jump height, reaction time, and flexibility. J Strength Cond Res. 2011;25(7):1925–31. 10.1519/JSC.0b013e3181e73959 21701282

[12] Stewart M, Adams R, Alonso A, Van Koesveld B, Campbell S. Warm-up or stretch as preparation for sprint performance? J Sci Med Sport. 2007;10(6):403–10. 17118704 10.1016/j.jsams.2006.10.001

[13] Behm DG, Chaouachi A. A review of the acute effects of static and dynamic stretching on performance. Eur J Appl Physiol. 2011;111(11):2633–51. 10.1007/s00421-011-1879-2 21373870

[14] Faigenbaum AD, McFarland JE, Schwerdtman JA, Ratamess NA, Kang J, Hoffman JR. Dynamic warm-up protocols, with and without a weighted vest, and fitness performance in high school female athletes. J Athl Train. 2006;41(4):357–63. 17273458 PMC1748418

[15] Gourgoulis V, Aggeloussis N, Kasimatis P, Mavromatis G, Garas A. Effect of a submaximal half-squats warm-up program on vertical jumping ability. J Strength Cond Res. 2003;17(2):342–4. 12741875 10.1519/1533-4287(2003)017<0342:eoashw>2.0.co;2

[16] Thompsen AG, Kackley T, Palumbo MA, Faigenbaum AD. Acute effects of different warm-up protocols with and without a weighted vest on jumping performance in athletic women. J Strength Cond Res. 2007;21(1):52–6. 17313270 10.1519/00124278-200702000-00010

[17] Turki O, Chaouachi A, Behm DG, Chtara H, Chtara M, Bishop D, et al The effect of warm-ups incorporating different volumes of dynamic stretching on 10-and 20-m sprint performance in highly trained male athletes. J Strength Cond Res. 2012;26(1):63–72. 10.1519/JSC.0b013e31821ef846 22158260

[18] Yamaguchi T, Ishii K, Yamanaka M, Yasuda K. Acute effects of dynamic stretching exercise on power output during concentric dynamic constant external resistance leg extension. J Strength Cond Res. 2007;21(4):1238–44. 18076260 10.1519/R-21366.1

[19] Tillin NA, Bishop D. Factors modulating post-activation potentiation and its effect on performance of subsequent explosive activities. Sports Med. 2009;39(2):147–66. 10.2165/00007256-200939020-00004 19203135

[20] Hodgson M, Docherty D, Robbins D. Post-activation potentiation: underlying physiology and implications for motor performance. Sports Med. 2005;35(7):585–95. 16026172 10.2165/00007256-200535070-00004

[21] Gullich A, Schmidtbleicher D. MVC-induced short-term potentiation of explosive force. New Stud Athletics 1996;11(4):67–81.

[22] Chattong C, Brown LE, Coburn JW, Noffal GJ. Effect of a dynamic loaded warm-up on vertical jump performance. J Strength Cond Res. 2010;24(7):1751–4. 10.1519/JSC.0b013e3181ddf665 20512065

[23] Cilli M, Gelen E, Yildiz S, Saglam T, Camur M. Acute effects of a resisted dynamic warm-up protocol on jumping performance. Biol Sport. 2014;31(4):277–82. 10.5604/20831862.1120935 25435670 PMC4203844

[24] Reiman MP, Peintner AM, Boehner AL, Cameron CN, Murphy JR, Carter JW. Effects of dynamic warm-up with and without a weighted vest on lower extremity power performance of high school male athletes. J Strength Cond Res. 2010;24(12):3387–95. 10.1519/JSC.0b013e3181f159bd 21088550

[25] Chatzopoulos DE, Michailidis CJ, Giannakos AK, Alexiou KC, Patikas DA, Antonopoulos CB, et al Postactivation potentiation effects after heavy resistance exercise on running speed. J Strength Cond Res. 2007;21(4):1278–81. 18076255 10.1519/R-21276.1

[26] Fukutani A, Hirata K, Miyamoto N, Kanehisa H, Yanai T, Kawakami Y. Effect of conditioning contraction intensity on postactivation potentiation is muscle dependent. J Electromyogr Kinesiol. 2014;24(2):240–5. 10.1016/j.jelekin.2014.01.002 24485557

[27] Zois J, Bishop DJ, Ball K, Aughey RJ. High-intensity warm-ups elicit superior performance to a current soccer warm-up routine. J Sci Med Sport. 2011;14(6):522–8. 10.1016/j.jsams.2011.03.012 21907619

[28] Amiri-Khorasani M, Osman NAA, Yusof A. Acute effect of static and dynamic stretching on hip dynamic range of motion during instep kicking in professional soccer players. J Strength Cond Res. 2011;25(6):1647–52. 10.1519/JSC.0b013e3181db9f41 21358428

[29] Kovacs M. Dynamic stretching: The revolutionary new warm-up method to improve power, performance and range of motion: Ulysses Press; 2009.

[30] Fletcher IM, Monte-Colombo MM. An investigation into the effects of different warm-up modalities on specific motor skills related to soccer performance. J Strength Cond Res. 2010;24(8):2096–101. 10.1519/JSC.0b013e3181e312db 20634747

[31] Gelen E, Dede M, Bergun Meric Bingul CB, Aydin M. Acute Effects of Static Stretching, Dynamic Exercises, and High Volume Upper Extremity Plyometric Activity on Tennis Serve Performance. J Sports Sci Med. 2012;11(4):600 24150068 PMC3763304

[32] Girard O, Micallef J-P, Millet GP. Lower-limb activity during the power serve in tennis: effects of performance level. Med Sci Sports Exerc. 2005;37(6):1021–9. 15947729

[33] Paradisis GP, Pappas PT, Theodorou AS, Zacharogiannis EG, Skordilis EK, Smirniotou AS. Effects of Static and Dynamic Stretching on Sprint and Jump Performance in Boys and Girls. J Strength Cond Res. 2014;28(1):154–60. 10.1519/JSC.0b013e318295d2fb 23591944

[34] Behm DG, Plewe S, Grage P, Rabbani A, Beigi HT, Byrne JM, et al Relative static stretch-induced impairments and dynamic stretch-induced enhancements are similar in young and middle-aged men. Appl Physiol Nutr Metab. 2011;36(6):790–7. 10.1139/h11-107 22014144

[35] Avela J, Kyröläinen H, Komi PV. Altered reflex sensitivity after repeated and prolonged passive muscle stretching. J Appl Physiol. 1999;86(4):1283–91. 10194214 10.1152/jappl.1999.86.4.1283

[36] Bingul BM. The Optimal Waiting Time for Hamstring Peak Power after a Warm-Up Program with Static Stretching. Anthropologist. 2014;18(3):777–81.

[37] Fowles J, Sale D, MacDougall J. Reduced strength after passive stretch of the human plantarflexors. J Appl Physiol. 2000;89(3):1179–88. 10956367 10.1152/jappl.2000.89.3.1179

[38] Knudson DV, Noffal GJ, Bahamonde RE, Bauer JA, Blackwell JR. Stretching has no effect on tennis serve performance. J Strength Cond Res. 2004;18(3):654–6. 15320640 10.1519/13553.1

[39] Mizuno T, Matsumoto M, Umemura Y. Decrements in stiffness are restored within 10 min. Int J Sports Med. 2013;34(6):484–90. 10.1055/s-0032-1327655 23143704

[40] Brandenburg J, Pitney WA, Luebbers PE, Veera A, Czjaka A. Time course of changes in vertical-jumping ability after static stretching. Int J Sports Physiol Perform. 2007;2(2):170 19124904 10.1123/ijspp.2.2.170

[41] Fernandez-Fernandez J, Sanz-Rivas D, Mendez-Villanueva A. A review of the activity profile and physiological demands of tennis match play. Strength Cond J. 2009;31(4):15–26. 10.1519/JSC.0b013e318194208a 19197208

[42] International Tennis Federation (ITF). Injury prevention: warming up. Acceded 2015. Available: http://www.itftennis.com/scienceandmedicine/injury-clinic/injury-prevention/warming-up

[43] Knudson D, Noffal G. Time course of stretch-induced isometric strength deficits. Eur J Appl Physiol. 2005;94(3):348–51. 15711989 10.1007/s00421-004-1309-9

[44] Bosco C, Luhtanen P, Komi PV. A simple method for measurement of mechanical power in jumping. Eur J Appl Physiol. 1983;50:273–82. 10.1007/BF00422166 6681758

[45] Cronin JB, Hansen KT. Strength and power predictors of sports speed. J Strength Cond Res. 2005;19(2):349–57. 15903374 10.1519/14323.1

[46] Fernandez-Fernandez J, Ellenbecker T, Sanz-Rivas D, Ulbricht A, Ferrauti A. Effects of a 6-week junior tennis conditioning program on service velocity. J Sports Sci Med. 2013;12(2):232–9. 24149801 PMC3761833

[47] Hornery DJ, Farrow D, Mujika I, Young W. An integrated physiological and performance profile of professional tennis. Br J Sports Med. 2007;41(8):531–6. 17472999 10.1136/bjsm.2006.031351 PMC2465445

[48] Hopkins W, Marshall S, Batterham A, Hanin J. Progressive statistics for studies in sports medicine and exercise science. Med Sci Sports Exerc. 2009;41(1):3 10.1249/MSS.0b013e31818cb278 19092709

[49] Hopkins WG. Measures of reliability in sports medicine and science. Sports Med. 2000;30(1):1–15. 10907753 10.2165/00007256-200030010-00001

[50] Batterham AM, Hopkins WG. Making meaningful inferences about magnitudes. Int J Sports Physiol Perform. 2006;1(1):50–7. 19114737

[51] Cohen J. Statistical power analysis for the behavioral sciences: Routledge Academic; 2013.

[52] Fernandez-Fernandez J, Ulbricht A, Ferrauti A. Fitness testing of tennis players: How valuable is it?. Br J Sports Med. 2014;48(Suppl 1):22–31. 10.1136/bjsports-2013-093152 PMC3995228 24668375

[53] Young W, Behm D. Effects of running, static stretching and practice jumps on explosive force production and jumping performance. J Sports Med Phys Fitness. 2003;43(1):21–7. 12629458

[54] Avela J, Finni T, Liikavainio T, Niemelä E, Komi PV. Neural and mechanical responses of the triceps surae muscle group after 1 h of repeated fast passive stretches. J Appl Physiol. 2004;96(6):2325–32. 14966020 10.1152/japplphysiol.01010.2003

[55] Cramer JT, Housh TJ, Johnson GO, Miller JM, Coburn JW, Beck TW. Acute effects of static stretching on peak torque in women. J Strength Cond Res. 2004;18(2):236–41. 15142021 10.1519/R-13303.1

[56] Cramer JT, Beck TW, Housh TJ, Massey LL, Marek SM, Danglemeier S, et al Acute effects of static stretching on characteristics of the isokinetic angle–torque relationship, surface electromyography, and mechanomyography. J Sports Sci. 2007;25(6):687–98. 17454536 10.1080/02640410600818416

